# Why We Will Continue to Lose Our Battle with Cancers If We Do Not Stop Their Triggers from Environmental Pollution

**DOI:** 10.3390/ijerph18116107

**Published:** 2021-06-05

**Authors:** Roberto Cazzolla Gatti

**Affiliations:** 1 Konrad Lorenz Institute for Evolution and Cognition Research, 3400 Klosterneuburg, Austria; roberto.cazzolla-gatti@kli.ac.at; 2 Biological Institute, Tomsk State University, 634050 Tomsk, Russia

**Keywords:** cancer, environment, pollution, air, water, soil, carcinogens

## Abstract

Besides our current health concerns due to COVID-19, cancer is a longer-lasting and even more dramatic pandemic that affects almost a third of the human population worldwide. Most of the emphasis on its causes has been posed on genetic predisposition, chance, and wrong lifestyles (mainly, obesity and smoking). Moreover, our medical weapons against cancers have not improved too much during the last century, although research is in progress. Once diagnosed with a malignant tumour, we still rely on surgery, radiotherapy, and chemotherapy. The main problem is that we have focused on fighting a difficult battle instead of preventing it by controlling its triggers. Quite the opposite, our knowledge of the links between environmental pollution and cancer has surged from the 1980s. Carcinogens in water, air, and soil have continued to accumulate disproportionally and grow in number and dose, bringing us to today’s carnage. Here, a synthesis and critical review of the state of the knowledge of the links between cancer and environmental pollution in the three environmental compartments is provided, research gaps are briefly discussed, and some future directions are indicated. New evidence suggests that it is relevant to take into account not only the dose but also the time when we are exposed to carcinogens. The review ends by stressing that more dedication should be put into studying the environmental causes of cancers to prevent and avoid curing them, that the precautionary approach towards environmental pollutants must be much more reactionary, and that there is an urgent need to leave behind the outdated petrochemical-based industry and goods production.

## 1. Introduction: Prevent Cancers Instead of Fighting Them

In a time when we are all concerned about the risks posed to human health by an unprecedented pandemic [1], we risk forgetting and underestimating the death toll of an old and lasting (at least, since the Industrial age) pandemic that affects almost a third of the human population worldwide: cancer (or, better, cancers). Almost 10 million people die from cancers annually, with a never decreasing trend since the beginning of the 19th century [2]. Cancers are now considered the second leading cause of death after only cardiovascular diseases. There is a higher prevalence of cancers in higher-income countries, from approximately 6% (which is about 34,000 people) of the population in the US down to around 0.4% in the poorest countries [3]. Globally, breast cancer is the most prevalent form, followed by prostate, colon, rectum, cervical, pulmonary, uterine, stomach, bladder, and other forms [2].

Seen in this perspective, we are forced to consider cancer as a homicide. This is because humanity considers, with good reasons, the number of COVID-19 victims intolerable but seems to accept the premeditated murder committed by cancers because of the anonymity of its victims. This statement might seem too strong, a boutade. In fact, for every murder, there must be an assassin. However, in the case of cancers, we are well aware to often be the victim of ourselves, in a sort of mass suicide: lifestyle has much to be blamed for cancer mortality, with obesity, sedentary habits, alcoholism, and smoking contributing as supposed major factors [4,5]. Instead, lifestyle should be considered as an accessory of the main killer: environmental pollution. For instance, lung cancer also heavily affects non-smokers and is the sixth most common cause of cancer-related death (18,000–27,000 deaths each year are caused by lung cancer in people who are not active cigarette smokers, and 16,000 occur among lifelong non-smokers [6,7]. Similarly, obesity seems to contribute but does not directly cause cancers [8]. Yet, alcoholism is a relevant inductor of liver cancer onset, but is neither the only cause nor the main one [9].

What is, rather, emerging with the recent research in environmental health is that the exposure to a mixture of pollutants, favoured by wrong lifestyles, psychosocial stressors, and genetic susceptibility, significantly increases the risk of developing cancer [10,11]. Global patterns of cancer incidence confirm this evidence [12]. The deaths in the worst-polluted places on Earth [13] are unequivocal proof. However, almost no place can be considered safe nowadays on this planet, with an even higher risk in industrialised countries. Occupational exposures to certain chemicals increase the incidence [14], but about 220 widely distributed breast cancer carcinogens have been identified so far [15,16], and we have increasing evidence that most children’s cancers are strongly related to parental exposures [17,18].

Our medical weapons against cancers have not improved too much in the last century, at least not in terms of the number of arrows in our quiver: once diagnosed with a malignant tumour, we still rely on surgery, radiotherapy, and chemotherapy, hoping to not discover a metastasis after some years. Then, no arrows remain. Quite the opposite, our knowledge of the links between environmental pollution and cancer has surged from the 1980s. Researchers started discovering the dangers of indoor pollution from domestic chemicals of common use, some in the past and most in the present, such as phthalates (contained in toys, personal care products, vinyl flooring and wall covering, detergents, lubricating oils, food packaging, pharmaceuticals, etc.) that impair male development and pose a risk to human reproduction [19], PCBs (polychlorinated biphenyls, widely used in electrical equipment like capacitors and transformers) that reduce testosterone [20,21], PFAS (perfluoroalkyl and polyfluoroalkyl substances, a group of ~5000 synthetic chemicals in commercial production since the 1940s to make surfaces resist stains, water, and grease, where the most widely studied are PFOA used for decades to make non-stick pans) that can cause testicular and kidney cancer [22], flame retardants (present in manufactured materials, such as plastics and textiles, and surface finishes), and bisphenol A (found even in polycarbonate baby bottles and the epoxy lining of food cans), leached in most food items [23,24,25] that, in laboratories, induced precancerous lesions to form in the mammary and prostate glands in early life [26,27,28,29].

Nonetheless, outdoor exposure to chemicals, particularly those used in agriculture, soon emerged as a major threat to human health. DDT (dichlorodiphenyltrichloroethane), a now-banned organochlorine insecticide in most countries, for a long time has been one of the most common pesticides found in fishes [30] (which also contain PCBs [31], and heavy metals [32]), in the bodies of migrating songbirds [33], in forest soils [34], in paints [35], in breast milk [36,37], and as residues on kitchen floors [38], together with other pesticides such as chlordane, chlorpyrifos, diazinon, fipronil, and permethrin. Close to 25 different insecticides can be detected in house dust [39]. Moreover, hormonal effects (which can stimulate mammary and ovary cancer) of atrazine exposure were shown by many early studies [40,41,42,43,44,45,46]. However, this pesticide was banned in Europe only in 2004, and is still allowed in the USA and some other countries [47]. Instead, very early on—already in the 1980s—the scientific community launched an alarm on the evidence that women with breast cancer have higher levels of DDE (a common breakdown product of DDT found in vertebrate’s bodies) and PCBs in their tumours [48], which was clearly linked to the increase of breast cancer among women born between 1947 and 1958 [49].

Unfortunately, all this preliminary scientific evidence has not always been followed by governments and institutions, which still fail to pursue research on cancer’s environmental connections [50]. Nature (see for instance whales with bladder cancer in [51] and even our companion animals were revealing a truth that we have long ignored. Continuous studies, from 1938 up to now, suggested the evidence that exposure to herbicides, insecticide, and waste pollutants poses a risk of cancer, particularly of the bladder, to dogs [52,53,54,55,56,57]. Yet, the coincident rise of synthetic dyes and bladder cancer among textile workers [58] and the growing evidence of higher bladder cancer risk for workers in rubber and metal industries [59,60,61] did not do much to address socio-economic and political actions.

On the contrary, and besides some sporadic bans, carcinogens in water, air, and soil have continued to accumulate disproportionally and grow in number and dose, bringing us to today’s carnage. We focus on fighting a difficult battle instead of preventing it by controlling its triggers. The following sections report the main findings of a literature review that was conducted, following the guidelines of the PRISMA [62] and the COSMOS-E [63] approaches, on studies publicly available as of 28 February 2021 and reporting on the links between exposure to environmental pollutants and cancer. PubMed/Medline, Web of Sciences (WoS), Scopus, and Google Scholar were searched. The inclusion criteria were the following: studies involving human participants and laboratory/domestic animal species (with some selected wild animal species exposed to anthropogenic pollutants), original research paper evaluating the association among environmental pollutants and cancer, or original research paper that reported at least one environmental pollutant as a potential cause of cancer. The exclusion criteria were the following: studies involving non-human and non-animal species, and publications containing editorials, letters to editor, case reports, and case series. In addition, non-English papers were also excluded. The first search identified ≈950 papers, where ≈650 were excluded after the abstract screening because they did not match the inclusion criteria. Therefore, ≈300 full-text articles have been assessed, of which ≈280 have been included in this review (Supplementary Figure S1). However, because this is not a fully comprehensive systematic review of the literature but, rather, a critical review of the state of the knowledge of the links between cancer and environmental pollution in the three compartments of air, water, and soil, here, a synthesis of the most relevant findings is provided and research gaps are briefly discussed, and some future directions are indicated.

## 2. Carcinogens into Water

So far, we have found, in water, about 35 of the 202 identified mammary carcinogens, including the triazine herbicides atrazine and symazine, acrylamide, DBCP, 1,2 dibromoethane, 1,2-dichloropropane, 1,2-dichloroethane, benzene, carbon tetrachloride, 3,3-dimethyoxybenzidine, styrene, and vinyl chloride [15]. In fact, most of the evidence about the cancerogenic effects of solvents and pesticides were brought to attention by the studies on contaminated drinking water [30,64]. Aquifers, lakes, and streams from which drinking water is taken can contain contaminants from personal care products, pharmaceuticals, hormones, and organic wastewater, which alter the endocrine system and, in turn, favour tumours [65,66,67].

Not only evidently problematic contaminants, such as pesticides [68,69]; see also “Carcinogens into Soil,” Section 4), metal degreasers, and dry-cleaning fluids [70] can be found in drinking waters, but even the abused nitrates in agriculture that leach in the aquifers can pose serious risks of developing cancer [71,72].

Paradoxically, drinking is not the only way to take in indoor carcinogens that are in outdoor water: dermal and inhalation routes of exposure have been shown as relevant sources of contamination due to volatilisation of chemicals [73,74,75,76]. These additional ways to come in contact with carcinogens from waters represent a serious danger to women and their infants who, for instance, during bathing and showering may be exposed to substances such as chloroform and trihalomethane, which increase the risk of developing tumours [75,77].

From the 1980s up to now, several studies have been conducted that show the link between contaminants in drinking water and cancer [78,79,80,81,82,83,84], including leukaemia induced by dissolved radioisotopes [85,86,87]. Heavy metal pollution in drinking water constitutes another serious risk for human health and is associated with many cancer forms [88,89,90]. Chromium and arsenic, which can naturally be part of some soils, but are in high levels in industrially contaminated groundwaters, are of serious concern for bladder, prostate, and kidney cancers’ induction, even at low–moderate concentrations [91,92,93]. High concentrations of arsenic in drinking water cause chronic intoxication that may sometimes also result in the development of skin and lung cancer [94,95]. At the same time, even though the small amount of chlorine used for tap-water disinfection is of no concern for human health [96], there still exists a link between water chlorination and bladder and other cancers [15,97,98], which seems to be due to the presence in source waters of other organic contaminants that can create by-products, such as the carcinogen MX [99].

Radioactivity from natural sources (e.g., uranium and dissolved radon in some rocky soils) and human activities (contamination from nuclear power plants, nuclear wastes, etc.) can be relatively high in drinking water and increase the risk of different cancer types, particularly of the digestive system [100,101,102]. However, most of these carcinogens are not currently checked for in routine and periodic controls of drinking waters made by local and national sanitary authorities.

## 3. Carcinogens into Air

The presence of carcinogens in the air is one of the most well-documented, because there is wide experimental evidence for the carcinogenicity of air pollutants [103]. There is, for instance, ample documentation of airborne carcinogens from industrial sites, road traffic, intensive farms, military bases, etc. [104,105]. Among air pollutants that may act as carcinogens, ultrafine particles are of higher concern because of their ability to penetrate deeply into the respiratory system [106,107,108].

Unfortunately, as in other compartments, most pollutants in the air—which may not be so cancerogenic in isolation—can have interactive effects [109]. An example is ozone [110,111,112], that can be formed by nitrogen oxides, emitted mainly from internal combustion engines, and separated into nitric oxide (NO) and free atoms of oxygen (O), by the visible or ultraviolet energy of sunlight. When the free atoms of oxygen produced by NO_x_ combine with molecular oxygen (O_2_), they form ozone (O_3_). In the presence of hydrocarbons, volatile organic compounds, and sunlight, these chemical reactions can take place and form photochemical smog [113], with secondary pollutants such as peroxyacetyl nitrates (PANs). These substances are considered “urban factors” in triggering lung cancer [114,115,116,117,118]. Several epidemiological studies have evidenced an increasing incidence of lung adenocarcinoma [119,120,121], a strong association between air pollution and cancer mortality [122,123], a correlation between industrial air pollution and cancer [124,125,126,127], and occupational cancer [14,128].

However, carcinogens in the air do not only trigger lung cancers but are documented risk factors for breast and bladder cancers [129,130,131]. Residence near industries and high traffic areas increases the risk of breast cancer [132,133], also because aromatic hydrocarbons, like the benzo[a]pyrene, which are emitted by the combustion of coal and petroleum derivates, are involved in the DNA mutagenesis of the mammary gland cells [134,135]. Similarly, bladder cancer is linked to air pollution, with a strong association of mortality among people living in residential districts polluted by industrial petrochemical plants [136,137,138,139].

Besides road traffic and industries, garbage management can also pollute the air and not only the more obvious soil and water compartments. Waste incinerators, in fact, may release dioxin [140,141], which can be harmful even in trace amounts [142] and is considered the most potent carcinogen because it is able to induce cancer in laboratory animals even at extremely low concentrations [143]. Despite recent progress in incinerator technology to reduce air pollutants, including dioxin and particulate (e.g., with “flameless” chambers, oxy-combustors, and molecular dissociation processes), what is ideally removed from air contaminants is, inevitably, concentrated in the residual fly ashes [144,145,146,147], which can be even more dangerous when stocked or dispersed into soil and water, even if vitrified [148]. Moreover, although modern technologies could reduce dioxin emissions, it is difficult to completely block the formation of easily escaping ultrafine particles [149,150], which forms even more and smaller particles at the high temperature of new incinerators [151]. The apparent advantage of volume reduction compared to “cold processing and dumping” is outweighed by the air emission of greenhouse gasses (CO_2_, CH_4_, etc.) and the concentration of even more toxic contaminants in the residual ashes that, however, must be stocked in special dumps. Additionally, when hospital waste is incinerated, it is one of the largest known sources of dioxin because it contains PVC (polyvinyl chloride) plastic, the dominant component of organically bound chlorine in waste such as bags, gloves, bedpans, tubing, and packaging [152].

Unfortunately, incineration is not the only source of dioxin: domestic fireplaces, firewood heating, kitchen chimneys, and agricultural controlled fires are also main sources [153,154]. It is, therefore, easy to understand that through air–water–soil pathway contamination, food may become a source of dioxin [155]. Chlorinated dibenzo-p-dioxins (CDDs) have been found in cow’s milk near incinerators [156,157], in rivers, fish, soil, and crops [142]. In several human and laboratory studies, dioxin showed the ability to induce liver and lung cancers [158,159], affect hormone production and growth factors [160,161], and act as a developmental toxicant [143,162,163]. It is becoming clearer that dioxin targets P450 enzymes and aryl hydrocarbon receptors (AhRs) in the human body, particularly during pregnancy, and this alters mammary epithelial cell proliferation and differentiation [159,164,165,166,167].

Of no less importance, and deserving at least a mention, are other sources of carcinogens in the air. One is asbestos, whose exposure is almost exclusively the cause of malignant mesothelioma, a cancer of the membranes surrounding the lungs [168]. Another, jet fuels, which—besides fine particles and other pollutants—contain a high level of octane boosters to improve the acceleration of aircraft, such as toluene, that produces, by nitration, a mixture of nitrotoluenes in exhaust fuels [169]. One of them, dinitrotoluene, which is also a military chemical propellant and explosive, is considered a probable carcinogen because it is linked with breast, liver, and bladder cancers, may pose a serious risk to people living near military bases [129,170,171,172,173]. Other nitrification products, such as the o-toluidine also deriving from the manufacture of dye-stuffs and the production of rubber, chemicals, and pesticides, have shown carcinogenic properties [174,175] and can pose another risk to exposed populations living close to airports and factories that emit toluidine. 

An additional source of carcinogenicity in the air may be electromagnetic fields and indoor radon. Some evidence is emerging about the risk posed by electricity, communication, and wireless devices [176,177,178], and there is strong evidence for an association between leukaemia, breast, and brain cancers and residential or occupational exposure to electromagnetic low frequencies, including cell phones [179,180,181,182,183], even if further research is needed to better clarify the effects on human health of the new technologies for smartphones and 5G [184,185,186]. Indoor radon—which accumulates in closed spaces from the natural radioactive decay of uranium of rocks in the building foundations and construction materials, producing radioactive particles that can be deposited on the cells lining the airways—may damage DNA and cause lung cancer [187,188].

## 4. Carcinogens into Soil

The research history of carcinogens into soil can be dated back to the discovery of the links between bladder cancer and aromatic amines in the 1970s–1980s. In fact, their evidence as harmful chemicals for human health forced authorities to ban a few of them and regulate their use in the workplace. The proof of their carcinogenicity came after some aromatic amines were removed from the chemical industry and the incidence of bladder cancer among affected workers declined considerably [189]. Although aromatic amines are a wide group of chemicals that include ingredients in tobacco smoke, most of their dissemination has been through pesticides in agriculture. Bladder cancer is common among farmers [190] and several aromatic amines, some of which are already banned in many countries (such as atrazine in Europe, but not in the USA), have been proven responsible for this. Atrazine was found in water before its ban and can even be detected in traces now, many years after its disappearance from agriculture. In the United States, where atrazine is still in use, it is contaminating aquifers and drinking water [69]. Soils contaminated by atrazine reached even freshwater animals, such as frogs [191].

This chemical component of some pesticides is a proven endocrine disruptor, which affects the hypothalamic control of pituitary-ovarian function [43,192] and triggers human ovarian cancers [193,194,195,196,197,198]. Moreover, atrazine impacts breast development and shows adverse effects of prenatal exposure during a critical period of mammary gland growth [42,162,199,200]. Despite that other human studies are needed to understand the effects of this contaminant on early-life exposure, there is not much doubt about its carcinogenicity for exposed people [196,201] and persistence in the environment [202]. 

Three other classic examples of persistent and dangerous pesticides are endosulfan, lindane, and parathion. Endosulfan is an organochlorine insecticide and acaricide chemically similar to DDT, largely sprayed on vegetables, apples, melons, and cotton for decades, which is being banned around the world only in recent years (in the USA only in 2010), although the evidence of its extreme risk is much older [203,204]. Lindane was widely used in the commercial tree industry but is being banned globally under the Stockholm Convention on Persistent Organic Pollutants, an international treaty negotiated at the United Nations Environment Programme (UNEP). Parathion was banned because of its high toxicity in the 1980s [205]. All of these three insecticides, particularly in occupational exposure, showed links with cancer [206,207].

Several other confirmed or potential carcinogens have been identified in pesticides [208,209,210]. Aldrin and dieldrin were used extensively in agriculture for at least two decades until their use was suspended in the 1970s in most countries, though these insecticides were being manufactured in many European countries at least until 1978 and are still found in some parts of the world [211]. Aldrin converts to dieldrin, which is considered one of the most persistent of all pesticides. Dieldrin remains in the environment for a long time and is usually detected in soil, sediment, and animal fatty tissues. Levels of both of these pesticides have decreased over the years since they are no longer produced or used, but some traces of them can still be found in soil and water [212]. Other agricultural chemicals have been associated with lymphomas and leukaemia. For instance, chlordane and heptachlor—banned in the 1980s in most countries—can increase the risk of developing non-Hodgkin’s lymphoma [213,214]. Today, these chemicals are still around us, since they can be found in soils and buildings long after treatment on various crops or insects was performed [215]. People were usually exposed to these chemicals by eating foods in which these substances accumulate, like those high in animal fat, such as meat, fish, and dairy products [216]. Pregnant women may have passed these chemicals to the foetus, and after birth through breast milk [217].

Unfortunately, the vertical mother–foetus passage of carcinogens is not isolated to chlordane and heptachlor but is quite common to most pesticides and can explain childhood and early-life cancers, particularly brain cancer and leukaemia [218,219,220,221,222]. Not only is contaminated food involved in this passage, but also exposition to pesticides in the homes of farmworker children plays a role [223,224]. Pesticides have been found even in carpet fibres of children of parents using pesticides outside [225,226]. However, also for women themselves, a new concern is emerging on the link between pesticide use and breast cancer risk [227,228,229,230].

As stated above, not all and not everywhere have carcinogen pesticides, which can accumulate into soil, and then leach into water and vaporise into air, been banned. For instance, although the European Union blocked atrazine from the market in 2004, the American EPA’s review on its safety remains controversial and has been criticised. From the 1980s, the EU started to implement some bans on other specific pesticides, mainly persistent organochlorine compounds, due to growing evidence of human or environmental harm. For example, the decision to ban the organochlorine insecticide DDT was made in 1986. The United States banned its use in 1972, but DDT is still manufactured in North Korea, India, and China. India is the largest consumer of the product for agricultural disease control. Similarly, 80–90% of DDT produced in China is used to synthesise Dicofol, an acaricide used to protect plants from pests. Most African countries do not use this chemical for agricultural purposes, but in countries such as Ethiopia, South Africa, Uganda, and Swaziland, DDT is still used to control malaria. Apart from people living in producing and employing countries [231], this may pose a risk to other populations around the world through exported food and goods in a global market [232].

Other, still-in-use potential carcinogens from pesticides are accumulating into soil worldwide. One of them, glyphosate, has been shown to cause harm in large doses [233]. The World Health Organization’s International Agency for Research on Cancer declared that it “probably” causes cancer [234], although the EPA and Bayer, the company that now owns the producer Monsanto, maintain that glyphosate does not cause cancer in humans [235]. Nonetheless, recent studies found a compelling link between exposures to glyphosate-based herbicides and increased risk for non-Hodgkin’s lymphoma and breast cancer [236,237].

Among other suspected carcinogens into soil, nitrates, which accumulate after over-fertilisation in agricultural products and leaches in aquifers, may increase the risk of bladder cancer [238,239,240,241]. Illegal disposal of garbage and even legal landfills are another source of carcinogens’ percolation and soil contamination [242]. The risk increases when populations are exposed to illegal dumping of toxic wastes and their burning [243]. 

## 5. A Matter of Dose and Time

It was in 2007 when about 200 environmental scientists signed a declaration, known as The Faroes Statement, to highlight evidence of the link between low-level exposures to common environmental chemicals during early life (as a foetus and in infancy) and following risks of health problems, including cancer, during adulthood [244]. The point was, and still is, that we need a shift from the concept that only the dose makes the poison to the compelling evidence that also the timing of exposure to carcinogens makes the poison [245].

We now know with more confidence that chemicals can alter breast development in early life [162,199], and that children are at greater risk due to their higher susceptibility, particularly to endocrine disruptors, and parents in contact with carcinogens [18,246,247,248]. Childhood cancers are becoming an issue of even more concern among oncologists and environmental biologists because the likelihood to develop an early-life tumour is increasing worldwide and environmental pollution is the main suspect [ [117,249,250,251,252,253]. At the same time, for adult women, the percentage of the upsurge in breast cancer, which was initially attributed to earlier detection [254], has become another confirmation of an alarming situation when it appeared clear that the rise in this type of cancer predates mammography and an increased detection cannot account for the higher incidence [255,256,257].

Variation of breast cancer incidence taught us another lesson during recent years: a drop evidenced in the USA among women between 1999 and 2003 was restricted to oestrogen-dependent tumours, and this lent support to the connection between breast cancer and the environment [258,259,260]. Recalling the fact that many environmental pollutants (such as the pesticides endosulfan, toxaphene, and dieldrin) can mimic oestrogen activity and affect human oestrogen-sensitive cells, their ban can explain the pace of breast tumours some decades later [261,262]. Similarly, the percent of lung cancer mortality thought to be due to smoking cannot account for all non-smoking lung cancer deaths [263,264].

It should not be forgotten what we have known since Paracelsus’ age: the time of exposure to carcinogens is the emerging awareness, but the duration of this exposure certainly worsens the situation. For instance, several occupations when workers spend much of their life in contact with a specific pollutant or a mix of them have been associated with non-Hodgkin’s lymphoma [265]. Lymphomas are also more common in golf course superintendents, because to keep the grass clean and homogenous, there is the need to spray a high quantity of pesticides [266]. Leukaemia is more frequent in people working for or living in proximity of nuclear power plants [267,268,269,270,271]. However, even sporadic exposure to low doses of pesticides and polychlorinated biphenyls can increase the risk of non-Hodgkin’s lymphoma and leukaemia [214,272,273,274,275,276,277]. Even residential herbicide use is associated with a higher risk of non-Hodgkin’s lymphoma [278,279] and dogs can also be exposed and develop malignant lymphoma [280,281]. 

## 6. Conclusions: Future Research, Precautionary Principle, and Acceptable Risk

Nowadays, cancer is the leading cause of death in humans before they reach old age [2,282], and some specific, once rare, types connected to environmental and occupational contamination are increasing (e.g., testicular cancer [117], thyroid cancer [283], non-Hodgkin’s lymphoma [284], leukaemia [285], etc.). After about three decades of research from the first evidence of a link between environmental pollution and cancer in the 1980s, it is easy to feel that we are all, directly or indirectly, subject to an uncontrolled experiment. This makes human studies difficult because humanity may, at this point, lack unexposed controls, such as human beings who have never been in contact with environmental pollution. For instance, all of us are believed to carry detectable levels of carcinogens in our bodies [286]. However, time and dose really matter and the issue of whether some specific pollutants can favour human cancers is, nowadays, commonly investigated by research approaches that compare cancer incidence and mortality rates among groups of people highly, moderately, and lightly exposed [287]. Just looking at the environmental exposition to pollutants, it is possible to identify the sector of the population that may have been subjected, for a certain amount of time, to a heavy dose of the substances under investigation. Cancer incidence and mortality rates of the heavily exposed group are then compared to those of the general population, whose exposures may be shorter or happening at much lower levels. A higher incidence and mortality among heavily exposed groups represents strong evidence. Therefore, future environmental health and epidemiological studies should focus on comparison among populations and take into consideration that, all other things being equal, a positive trend in cancer rates within the people more exposed to potential sources of carcinogens would indicate a quite compelling confirmation of a risk for environmental contamination and human health. Although many animal and laboratory studies have paved the road for the discovery of the links between environmental contamination and cancers, interspecies differences in susceptibility [288,289] and the huge toll of sacrificed animals due to lab experiments [290] call for a paradigm shift towards human studies through the development of more centres for environmental oncology.

Taking into consideration that, although almost after a century of advanced research to find cures for tumours, the global cancer mortality rate has hardly decreased (even considering the ageing and a larger world population; [282], and that mortality rates are considered more reliable than incidence data in evaluating the trends [291], even more dedication should be put into studying the environmental causes of cancers to prevent and avoid having to cure them. In doing so, the precautionary approach towards environmental pollutants must be much more reactionary. Any unknown effect of released chemicals, even if they are not initially suspected as carcinogens, must be considered an unauthorised experiment on people who are involuntarily acting as guinea pigs and a new potential ecocide. 

There is, at the same time, an urgent need to leave behind the outdated petrochemical-based industry and goods production, which are the main sources of dangerous pollutants [292], and, simultaneously, the cause of global changes and biodiversity loss [293,294,295], and to move towards a carbohydrate-based economy [296]. This transition process has begun in some parts of the world, for instance with the replacement of petrochemical plastics with plant-derived disposable packaging, bottles, and shoppers [297,298,299], and with the increased production and consumption of more organic food, which avoids the use of pesticides and chemical fertilizers [300] (Hyland et al. 2019). 

It is a starting point, but if we want to win our battle with cancers, we need to stop all their triggers from environmental pollution.

## Supplementary Materials

The following are available online at https://www.mdpi.com/article/10.3390/ijerph18116107/s1, Figure S1: A flow diagram of the literature search methodology carried out following the guidelines of the PRISMA (Moher et al. 2009) and the COSMOS-E (Dekkers et al. 2019) approaches, on studies publicly available as of 28 February 2021.
