# Supplementary material for: Why We Will Continue to Lose Our Battle with Cancers If We Do Not Stop Their Triggers from Environmental Pollution
Source: Int J Environ Res Public Health. 2021 Jun 5;18(11):6107. doi: 10.3390/ijerph18116107 (PMC8201328; doi:10.3390/ijerph18116107)
Supplement: Supplementary file 1 [file ijerph-18-06107-s001.zip › ijerph-1157558-SI.pdf]

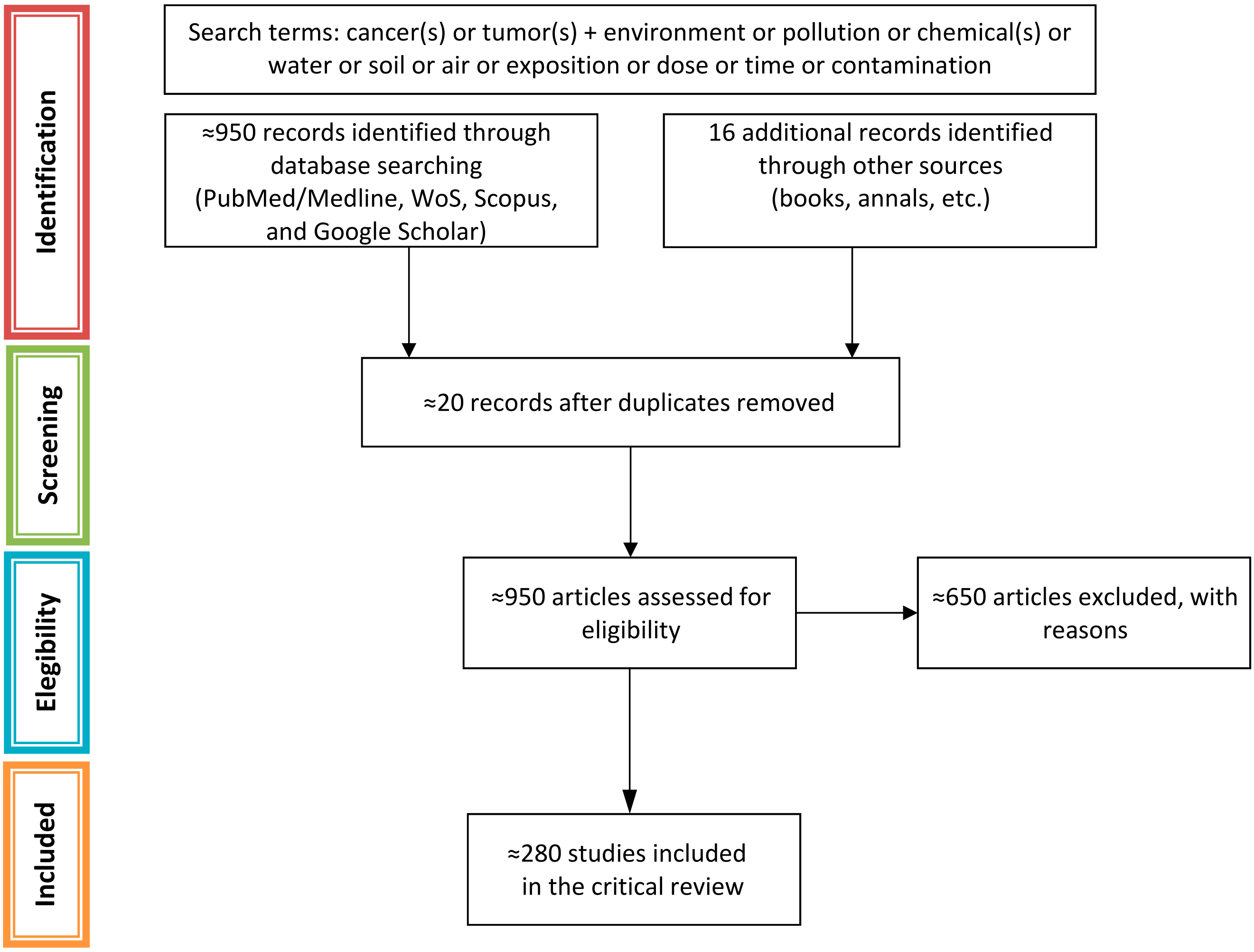

Supplementary Figure S1. A flow diagram of the literature search methodology carried out following the guidelines of the PRISMA (Moher et al. 2009) and the COSMOS-E (Dekkers et al. 2019) approaches, on studies publicly available on 28 February 2021
